# The Philosophical Foundations of a Multidisciplinary Approach to Psychiatry

**DOI:** 10.7759/cureus.80899

**Published:** 2025-03-20

**Authors:** José Carlos Medina-Rodríguez

**Affiliations:** 1 Research Promotion Unit, National Institute of Psychiatry Ramon de la Fuente Muñiz, Mexico City, MEX

**Keywords:** mental health, neuroethics, philosophy of mind, psychiatric diagnosis, psychiatry, transdisciplinary research

## Abstract

The continued growth of psychiatry requires ongoing dialogue and collaboration across varying disciplines to better understand the foundations of this discipline. As the world is changing in active and sometimes unexpected ways, the need for integrated and individualistic interventions has grown ever more important. Therefore, it is important to include professionals who study philosophy, neuroscience, psychology, sociology, and cultural analysis in modern psychiatric theoretical foundations. The above integrated approaches help to provide a more in-depth understanding of mental health and psychopathology. This means that medical specialists can approach classic and contemporary epistemological inquiries from both clinical and philosophical perspectives. For example, findings from neurobiology can address psychopathology from a positivist and logical angle. On the contrary, social and cultural assumptions suggest that individual and collective experiences affect mental health and psychopathology in varying degrees. Given the importance of this, researchers, clinicians, and philosophers could cooperate to develop hypotheses or principles that better explain the basic foundations of psychopathology and psychiatry. This joint effort allows psychiatric paradigms to be formulated in a broader, relativistic perspective that considers multicultural demographics and specific social contexts. Supporting the previous arguments, it is logical to place emphasis on the implementation of philosophical coursework in psychiatric fellowship programs. Philosophy tuition enables psychiatrists to develop solid thinking skills and scientific observation. However, it may be difficult to reinstate formerly consolidated but not currently integrated philosophical theory and logical empiricism into the medical community. Even so, the growth of psychiatry is contingent on intercollegiate activity that advocates crossover across different ideological branches. The previously stated goal places the mental health profession in more complex circumstances than those dealt with in the prior two centuries. Even with the previous hardships, this editorial aims to encourage psychiatrists to become increasingly involved regarding the proposed conceptual framework to further develop this unique specialty.

## Editorial

Introduction

Philosophical inquiries into human nature have extensively shaped the medical academic field since its inception. Before the philosopher Socrates, beliefs about the natural world did not emphasize the primordial attributes of the human psyche. Due to an absence of engagement toward inquiries about the properties of the mind, ideas were formed in favor of classical and formal logic. In the medieval era, these propositions were followed by changes that emphasized Christian theology, ethics, and moral reasoning. Over time, there was a concerted effort to adopt a more materialistic, logical-positivistic position in the natural sciences and, by extension, the medical field as an applied form of biology. However, the above assertions were soon followed by an entirely original argument about one of the foundations of the human mind and consciousness. The assertion was Cartesian dualism, introduced by René Descartes, who put forward the divisive mind-body problem, which was famously expressed in the Latin phrase, “Cogito, ergo sum.” Descartes' proposal laid the foundation for a description where mind and body were separated substances. However, he faced many counterclaims from both logical-empiricist and newer philosophical communities. However, this proposal later led to the birth of psychiatry, a medical specialty that used the constraints identified by neurologists to explain phenomena not directly related to traditional medical diagnostic methods. The adoption of these constraints helped the first psychiatrists freely explore the relationship between human physiology and the mind. These ideas eventually became the foundation for mental health, psychopathology, treatment, and later classification models. As expected, psychiatry faced many challenges as a newly developed medical specialty. Even though the first mental health professionals were motivated to study this field, they were inevitably challenged by the uncompromising nature of a profession that predominantly dealt with a limited amount of established, materialist, and medical philosophical thought. Due to this, more descriptive diagnostic models had to be made to distinguish between conditions that did not fit into traditional medical categories. These problems impacted the initial therapeutic interventions, which also failed to alleviate human psychiatric suffering because of a lack of understanding of intersecting ideologies, which, at the time, were mostly in conflict. Unexpectedly, medical innovations in the post-war II era serendipitously influenced psychiatry for the better. While these breakthroughs helped build a more successful psychiatric system, questions about the epistemology of psychopathology remained. Again, the hard inquiries posed by the aforementioned questions led to a reduced perspective about the human mind and its psychopathology to an even more descriptive model of the human mind. This approach, fueled by the cognitive revolution of the last century and discoveries in neurobiology, led psychiatrists to look for what they once embraced in opposition to the orthodox medical method: a complete reduction to materialist, logical, and positivist thought. In consequence, the scientific community rejected phenomenology (the study of subjectivity), existentialism (the inquiry into the incomprehensibility of existence), and hermeneutics (the study of meaning beyond linguistics). The above newly established and foundational disciplines were passed on to the social and cultural research communities. Even so, the prior disciplines would overcome disciplinary limitations if they were brought together and reintroduced to the medical sciences. In summary, these integrations have the potential to become an integral part of a rational and pragmatic method that may one day progress to a type of reasonable empiricism, as psychiatrists have sought to achieve for a century.

Objective

This editorial aims to advocate the proposal of cross-disciplinary psychiatry with a focus on philosophy; also, this proposal attempts to integrate logical empiricism and nonreductionist philosophical principles that contest the universality of society and culture in favor of a relativistic interpretation. By doing so, this proposal aims to align modern philosophy with advances in psychiatry to develop a more in-depth medical specialty while maintaining its unique position among the medical sciences.

Philosophy, logical positivism, and interpretation in psychiatry

Philosophy refers to the study of the nature of knowledge, reality, and existence. Meanwhile, psychiatry is a medical specialty that focuses on measurable, predictable, and reproducible causal relationships in mental health and psychopathology. As noted in the previous section of the editorial, the above approach emerged through the deconstruction of Renaissance and 19th- and 20th-century models that did not employ a materialist approach while not necessarily rejecting the scientific method. During the past 100 years, advances in neuroimaging, biomarker identification, and genomic research have encouraged logical-positivist thinking in the medical sciences. Questions were raised about whether psychiatry could adopt a similar framework and integrate with the rest of its medical brethren. Despite this, there remains a persistent limitation in that subjective experiences of the human mind and psychopathology resist narrowing themselves into full empiricism. This is due to factors such as personal experiences and their interpretive value, affective relevancy, and societal and cultural contexts. The above elements, inherent to humanity, invariably influence the ongoing research on diagnostic criteria and treatment protocols. However, they are recently becoming more flexible in their epistemological understanding and integration into the broader medical sciences. Examples of this advancement include the inclusion of transdiagnostic models, such as the Hierarchical Taxonomy of Psychopathology, which does not replace current classification models in psychiatry but encourages research in this field using a dimensional framework. This type of reasoning evaluates psychopathological characteristics across psychiatric disorders in a continuous and flexible manner. The previous statement is not a new one. In a nondichotomous format, philosophers such as Wilhelm Dilthey, Karl Jaspers, and Ludwig Binswanger addressed the mind-body problem, existential coherence, and meaning-making as central components of psychopathology. Similarly, Foucault [1] and Hacking [2] suggested an apparent interplay between psychiatric nosology, social group dynamics, cultural analysis, and personality formation, arguing that these processes may be neither entirely measurable nor systematically approachable but context-dependent. Consequently, the above inquiries have influenced current psychiatric classification models by leading to a more comprehensive appreciation of how societal and cultural factors shape diagnostic methods and treatment interventions relativistically. The acceptance of pragmatic variations of previous and recent epistemological methodologies has led to the cultivation of dynamic and constructive psychiatry. This assertion could provide a thorough and unbiased assessment of personal or communal experiences that could vary regarding distinct societies and cultures. Historically, psychiatric diagnoses have been changed in response to societal and cultural variables. These characteristics, which are validated and proven reliable, were laid out earlier by the above visionary figures. In accordance with the preceding statements, thought processes about psychiatry do gradually adapt to changing frames of reference, particularly regarding how psychopathology is expressed, understood, and perceived. Despite this, traditionalistic and intolerant behaviors continue to exist, sustaining attitudes that qualify as stigma, defined as societal prejudice and negative judgment of mental health as a whole. Even though the latter falls outside the scope of this editorial, the aforementioned assumptions have led to further research that emphasizes the call for unifying empirical reasoning with interpretative competence to develop more complete, multidisciplinary psychiatric models.

Integrating empiricism and societal and cultural analyses in psychiatric diagnosis

Psychiatry's pursuit of a universal diagnostic categorical model is influenced by cultural, historical, and medical values, which lead to a stance of superficial and nonreproducible empiricism. This position, which in part contradicts the scientific method, has led to thinking about novel modern classification systems that recognize that psychiatric specifiers, for example, deficits in prosociality, may be considered pathological in some cultures but not in others. For instance, Szasz's critique of psychiatric constructs [3] raised unexpected counterarguments, acknowledging the limitations of diagnostic classifications while addressing their potential merits but adjustable possible relativistic frameworks. Hence, current systems try to not only aim for descriptive, unequivocal diagnoses but also accept slowly the relativistic aspects of psychopathology. As such, present systems have trouble balancing social and cultural differences but do try to improve diagnostic criteria to favor a useful and imperfect broad validity. Yet, present models still have trouble balancing universality. As before, philosophers such as Kendler advocated for a pragmatic pluralistic theory, which defines diagnostic designations as provisional albeit meaningful since they translate to empirical interventions [4]. In a similar, relativistic vein, Frances suggests the merit of versatile reasoning to adjust to constantly shifting clinical discoveries in psychiatry, which, in turn, can affect sociocultural contexts. As this debate becomes more complicated, it will be important to remodel classification structures to encourage multidisciplinary innovations [5].

Philosophy of science and of the mind and its relevance to the epistemology of psychopathology

Cognitive science and philosophy added complexity to psychiatric theory by exploring consciousness, identity, and subjective experience beyond logical positivism and the aforementioned interpretive frameworks. Theories like eliminative materialism and higher order thought propose that consciousness and self-awareness come from material processes, making it harder to study these phenomena. These implications impact diagnostic models, blurring the lines between neurological dysfunction and experiential phenomena. The proposals also challenge disciplinary methods that are designed to balance empirical and philosophical considerations. Furthermore, recent advances in neuroscience have demonstrated that philosophical inquiry complements psychiatric progress, as seen in the historical integration of phenomenology and existential thought, but incompletely. For example, fields like neurophenomenology integrate subjective experiences with neurobiological data, which influences research on disorders that affect consciousness but also have conceptual limitations. However, prior discussions on emergent properties and mind-body relationships do acknowledge the varied nature of individuality and psychiatric disorders. This helps to refine diagnostic criteria and foster a nuanced view of psychiatry.

Closing remarks

Given the above epistemological constraints, neither reductionist nor purely interpretive models adequately capture the complexity of psychiatric disorders. Yet, combining empirical scientific validity with interpretive extent offers the birth of a pragmatic, realistic, and multidisciplinary conceptual framework that seems plausible and beneficial. Unlike strict reductionist models that exclude other nonmaterialist scientific methodologies, multidisciplinary approaches weave together philosophy, biology, psychiatry, and the social sciences. However, integrating different methodologies presents major epistemological and methodological questions, especially when trying to reconcile quantitative data with qualitative interpretation. These challenges have been either overlooked or inadequately addressed in psychiatric educational environments, curbing the acquisition of critical thought in an inherently eclectic field. For example, some psychiatric fellowship training programs prioritize conventional diagnostic criteria and pharmacological treatments while providing scant accessibility to the intellectual discourse on the philosophical foundation principles of mental health and psychopathology. Furthermore, trainees may lack the methodological means to comprehensively appraise emerging classification models, alternative management strategies, or the implications of neurobiological findings on psychiatric practice. This gap could be addressed by including the philosophy of science and of the mind and the previously mentioned disciplines in psychiatric education. However, historical constraints should be overcome, such as outdated educational systems and the prioritization of materialistic instruction. It is possible to overcome these barriers by integrating specialized philosophical and sociocultural coursework, as well as multidisciplinary workshops that involve scholars outside the boundaries of psychiatry. This may encourage constructive debate on the epistemology of modern diagnostic conceptual models and methodologies within residency programs, which may provide a crucial and yet diminishing skill in trainees: the cultivation of a solid and critical thought process. This may lead to a more effective management of patients and their clinical outcomes. Furthermore, this foundational competence is expected by virtue of being an applied scientific discipline born from time-tested philosophy. Therefore, acknowledging and addressing these barriers is necessary for promoting dialogue, improving methodological approaches, and bringing philosophical experts into psychiatric study environments to enrich the existing theoretical knowledge of mental health and psychopathology. Philosophers can help psychiatrists design procedures by changing hypothetical models that psychiatrists might overlook in routine research. This can help psychiatrists design procedures by revising hypothetical models that psychiatrists might miss in routine research. Their participation could help bridge the gap between empirical findings and broader ideological perspectives, leading to improved multidisciplinary and integrative psychiatric frameworks.

Conclusions

By comprehensively revisiting its philosophical core principles, psychiatry can benefit from a reformulation of its epistemological foundations. Redefining the field's empirical and philosophical principles may be able to enhance the validity of existing and emerging diagnostic frameworks by enhancing the validity of existing and emerging diagnostic frameworks. Rethinking assumptions can lead to more rational and realistic models of mental health and psychopathology, which can lead to more rational and realistic models of mental health and psychopathology. Therefore, it is important to develop a multidisciplinary approach to mental healthcare that includes other disciplines and professionals like philosophers. This partnership could be achieved by establishing philosophical training programs in psychiatric educational institutions, encouraging scholarly collaborations, and implementing philosophical principles in research and clinical psychiatric studies. Ultimately, these steps could improve the field's theoretical coherence, improve diagnostic models, and ultimately lead to better patient-centered approaches to mental health practice.

## References

[1] Foucault M (1965). Madness and Civilization: A History of Insanity in the Age of Reason.

[2] Hacking I (1995). Rewriting the Soul: Multiple Personality and the Sciences of Memory. https://press.princeton.edu/books/paperback/9780691059082/rewriting-the-soul?srsltid=AfmBOorLso008nGC0zxzRVLS8Zq18HN8jw5BLaJ_Mq8hc-xQmLfT8ACT.

[3] Szasz T (1960). The myth of mental illness. Am Psychol.

[4] Kendler KS (2016). The nature of psychiatric disorders. World Psychiatry.

[5] Frances A (2013). The past, present and future of psychiatric diagnosis. World Psychiatry.

